# Rare Detection of *Bordetella pertussis* Pertactin-Deficient Strains in Argentina

**DOI:** 10.3201/eid2511.190329

**Published:** 2019-11

**Authors:** Francisco Carriquiriborde, Victoria Regidor, Pablo M. Aispuro, Gabrielli Magali, Erika Bartel, Daniela Bottero, Daniela Hozbor

**Affiliations:** Universidad Nacional de La Plata y Consejo Nacional de Investigaciones Científicas y Técnicas, La Plata, Argentina

**Keywords:** pertussis, Bordetella pertussis, bacteria, pertactin, pertactin-deficient strains, vaccines, resurgence, vaccination, acellular vaccine, whole-cell vaccine, primary vaccination series, Argentina

## Abstract

Pertussis resurgence had been attributed to waning vaccine immunity and *Bordetella pertussis* adaptation to escape vaccine-induced immunity. Circulating bacteria differ genotypically from strains used in production of pertussis vaccine. Pertactin-deficient strains are highly prevalent in countries that use acellular vaccine (aP), suggesting strong aP-imposed selection of circulating bacteria. To corroborate this hypothesis, systematic studies on pertactin prevalence of infection in countries using whole-cell vaccine are needed. We provide pertussis epidemiologic data and molecular characterization of *B. pertussis* isolates from Buenos Aires, Argentina, during 2000–2017. This area used primary vaccination with whole-cell vaccine. Since 2002, pertussis case incidences increased at regular 4-year outbreaks; most cases were in infants <1 year of age. Of the *B. pertussis* isolates analyzed, 90.6% (317/350) contained the *ptx*P3-*ptx*A1-*prn*2-*fim*3-2 allelic profile. Immunoblotting and sequencing techniques detected only the 2 pertactin-deficient isolates. The low prevalence of pertactin-deficient strains in Argentina suggests that loss of pertactin gene expression might be driven by aP vaccine.

Vaccination against pertussis is mandatory worldwide. Two types of vaccines are currently in use: whole-cell vaccine (wP), which was the first vaccine developed, and acellular vaccine (aP), which contains purified components of *Bordetella pertussis* and was formulated because of adverse reactions associated with wP (*1*). Many countries continue to use wP for the primary vaccination series and for boosters recommended for children <7 years of age. Industrialized countries have switched to vaccination with aP. However, in the past 2 decades, the number of pertussis cases detected increased to ≈24.1 million/year; ≈161,000 deaths occurred during 2014 (*2*,*3*). Although most cases occur in developing countries, industrialized countries have also had large-scale outbreaks, even nations with high vaccination rates (*2*,*4*–*6*).

The main causes proposed for this changing pertussis epidemiology are vaccination coverage rates lower than the 90% recommended by the World Health Organization, waning of vaccine-induced immunity (*7*,*8*) (which occurs faster in the aP-vaccinated population), and evolution of circulating bacteria to vaccine immunity–evasive phenotypes (*9*,*10*). The first reports on bacterial evolution documented genetic polymorphisms encoding proteins included in the vaccines (e.g., pertactin [PRN], pertussis toxin, and the pertussis toxin promoter [*ptx*P]) (*11*,*12*). More recently, a major increase in the isolation of *B. pertussis* bacteria that do not express certain vaccine antigens was reported (*10*,*13*,*14*). In countries using PRN-containing aP vaccines, such as the United States, Canada, and Australia, the PRN-deficient isolates have increased substantially in the past 4 years (*10*,*15*,*16*). The expansion of strains deficient in PRN in populations vaccinated with PRN-containing aP vaccines indicates that such strains apparently have a selective advantage in populations vaccinated with aP vaccine (*17*).

To corroborate this hypothesis, we conducted systematic studies on PRN prevalence in Argentina, a country that uses wP vaccine. We monitored and analyzed *B. pertussis* population dynamics in Buenos Aires. Our aim was to assess whether PRN-deficient strains were circulating in Buenos Aires and to analyze the results obtained in relation to the vaccine used and the epidemiologic situation of the disease during 2000–2017.

## Materials and Methods

### Population Studied, Clinical Case Definition, and Laboratory Diagnosis

We used pertussis epidemiologic data and samples collected during 2000–2017 from the Pertussis Reference Laboratory (La Plata, Argentina). We collected data on patient sex, age, duration of symptoms, vaccination status, and laboratory results.

We confirmed clinical cases of pertussis in patients by isolation of *B. pertussis* culture, amplification of *B. pertussis*–specific DNA by using PCR, or serologic analysis (IgG titer to pertussis toxin >120 IU/mL). We defined a confirmed case of pertussis as one that meets the clinical case definition and is epidemiologically linked to a laboratory-confirmed case (*18**–**20*).

### Vaccine Schedule in Buenos Aires

The wP vaccine was introduced in Argentina (population 44.9 million) during the 1970s and is still used for the 3 primary doses at 2, 4, and 6 months of age; for the 2 boosters at 18 months of age; and at school entry (5–6 years of age) in the public sector (≈90% of the population). The aP vaccine is used in the private sector and for the recommended boosters in adolescents, healthcare workers in contact with infants <12 months of age, household contacts of low-birthweight infants, and pregnant women.

In most of Argentina, the diphtheria–tetanus–pertussis vaccine is used as a third dose; coverage during recent years ranged from 91.0% to 95.0%; in certain jurisdictions, this value was <80.0% (*21*). Official coverage rates for adolescent boosters were 75.3% during 2015, 81.9% during 2016, and 88.0% during 2017. Official coverage rates for maternal immunization were 61.7% during 2015, 65.6% during 2016, and 67.0% during 2017.

### Samples and Bacterial Growth Conditions

The Pertussis Reference Laboratory samples included nasopharyngeal specimens from 16,151 hospitalized patients from Buenos Aires with signs/symptoms of pertussis infection. These samples were routinely screened for *B. pertussis* by culture and PCR. *B. pertussis* culture was performed on Regan-Lowe agar (Difco, https://www.fishersci.com) supplemented with 15% (vol/vol) defibrinated fresh sheep blood at 36.5°C and monitored for 10 days. Suspected colonies were replicated in Bordet-Gengou agar (Difco) supplemented with 15% (vol/vol) defibrinated fresh sheep blood. Colonies exhibiting hemolysis were gram-stained and tested by using agglutination with *B. pertussis*–specific antiserum (Murex Diagnostic Products, https:///www.murex-ph.com) and PCR (*22*,*23*). The isolates were also biochemically typed by using the API-20-NE system (bioMérieux, https://www.biomerieux.com).

Isolates were stored at –80°C in 1% (wt/vol) casaminoacid solution containing 20% (vol/vol) glycerol. *B. pertussis* strain Tohama phase I (Collection de l’Institut Pasteur) was also grown on Bordet-Gengou agar at 36.5°C for 72 h.

### *B. pertussis* Isolate Characterization

#### Genotyping

A total of 350 *B. pertussis* isolates collected in Buenos Aires during January 2000–December 2017 were included in the analyses (Table 1). For genetic typing, we amplified *ptx*P, pertussis toxin A subunit (*ptx*A), *prn*, and fimbriae type 3 (*fim*3) loci by using PCR with specific primers (Table 2) and sequenced them as described (*24*–*32*). We also screened isolates for an array of mutations causing deficiency in the immunogen PRN by using PCR amplification and molecular sequencing (*26*,*27*). We used primers 5′-CCCATTCTTCCCTGTTCCAT-3′ and 5′-CCTGAGCCTGGAGACTGG-3′ (*27*) to amplify the complete *prn* gene (*27*), and these primers in combination with internal primers to sequence the complete gene.

**Table 1:** Immunization status of patients infected with *Bordetella pertussis* strains studied, Argentina, 2000–2017*

Year	No. strains	Patient information						
		<2 mo of age	<6 mo of age		6–12 mo of age		>12 mo of age	
		Unvaccinated because of age	Incomplete vaccination schedule	Complete vaccination schedule	Incomplete vaccination schedule	Complete vaccination schedule	Incomplete vaccination schedule	Complete vaccination schedule
2000	7	3	1	1	2	0	0	0
2001	7	2	1	1	3	0	0	0
2002	5	3	1	1	0	0	0	0
2003	9	4	2	2	1	0	0	0
2004	9	3	2	2	2	0	0	0
2005	6	3	1	1	1	0	0	0
2006	6	0	3	0	3	0	0	0
2007	38	10	10	9	6	3	0	0
2008	45	21	10	6	4	0	4	0
2009	7	3	2	2	0	0	0	0
2010	6	4	2	0	0	0	0	0
2011	86	40	20	16	2	4	0	4
2012	59	20	15	10	0	9	5	0
2013	6	3	0	0	0	3	0	0
2014	3	0	2	0	0	1	0	0
2015	6	3	1	1	0	0	1	0
2016	32	12	1	8	2	0	1	8
2017	13	3	1	1	0	2	0	6

*Complete vaccination schedule indicates that a person according to his or her age received the total number of doses indicated in the National Vaccination Calendar. Incomplete vaccination schedule indicates that a person according to his or her age did not receive the total number of doses indicated in the National Vaccine Calendar.

**Table 2:** Primers used in PCR for *Bordetella pertussis* strains studied, Argentina, 2000–2017*

Gene†	Primer sequence, 5′→3′	Reference(s)
*ptx*P	F: AATCGTCCTGCTCAACCGCC	(*27**,**28*)
	R: GGTATACGGTGGCGGGAGGA	
*ptx*A	F: CCCCTGCCATGGTGTGATC	(*29*)
	R: TCAATTACCGGAGTTGGGCG	
*prn*	F: CAATGTCACGGTCCAA	(*26*)
	R: GCAAGGTGATCGACAGGG	
*fim3*	F: GACCTGATATTCTGATGCCG	(*31*)
	R: AAGGCTTGCCGGTTTTTTTTGG	

*F, forward; R, reverse. †*fim*3, fimbriae type 3; *prn*, pertactin; *ptx*A, pertussis toxin subunit A, *ptx*P, pertussis toxin promoter.

We calculated by year the discriminatory power of the multilocus sequence typing technique by using the equation reported by Hunter and Gaston (*33*). This equation is based on the probability that 2 unrelated strains sampled from the test population will be placed into different typing groups. Thus, the index can have any value between 0 and 1, with 0 representing the lowest discriminatory capacity, indicating that all the strains are in a single genotyping group (low diversity), and 1 representing the highest discriminatory capacity, indicating high genotypic diversity among the isolates.

#### PRN Immunoblotting

For this assay, we treated 2 ×10^10^ CFUs of *B. pertussis* isolates with Laemmli sample buffer, and subjected extracts to electrophoresis on 12.5% (wt/vol) sodium dodecyl sulfate–polyacrylamide gels. After electrophoresis, we transferred the proteins to a polyvinylidene phosphate membrane (Immobilon P; Millipore, http://www.emdmillipore.com) and incubated with a 1:2,500 dilution of PRN-specific polyclonal immune serum. This serum was obtained from BALB/c mice immunized with purified *B. pertussis* 69-kDa PRN (National Institute for Biological Standards and Control [NIBSC] code no. 90/654 version 4). We used alkaline phosphatase–labeled sheep anti-mouse immunoglobulins for detecting immune complexes and nitroblue tetrazolium and 5-bromo-4-chloro-3-indolyl phosphate as phosphatase chromogenic substrates (Biodynamics SRL, https://www.biodynamics.com.ar). The *B. pertussis* Tohama strain served as a PRN-positive control.

#### Serotype Analysis

We performed serotype analysis by using an agglutination assay with monoclonal antibodies (mABs) against fimbriae type 2 (anti-Fim2 mAb; NIBSC code no. 04/154) and fimbriae type 3 (anti-Fim3 mAb, NIBSC code no. 04/156). These analyses followed European Union laboratory group recommendations (*34*).

## Results

### Pertussis Epidemiology in Buenos Aires

During 2000–2017, the Pertussis Reference Laboratory received 75% of total clinical samples (nasopharyngeal swab samples) from pertussis-suspected case-patients identified in Buenos Aires and reported to the Ministry of Health. Of these 16,151 samples, 3,220 (19.9%) were from laboratory-confirmed cases. A total of 2,870 samples were positive by PCR for *B.* pertussis–specific genes, and 350 samples were positive by PCR and culture.

**Figure 1:**
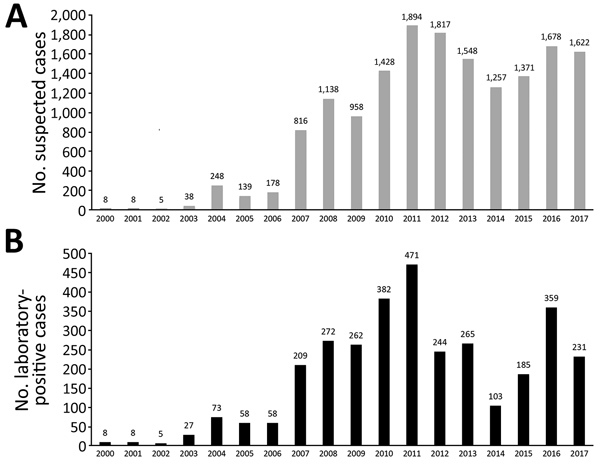
Pertussis cases per year reported to the Pertussis Reference Laboratory, Buenos Aires, Argentina, 2000–2017. A) No. suspected cases. B) No. laboratory-positive cases. Numbers above the bars indicate actual values.

**Figure 2:**
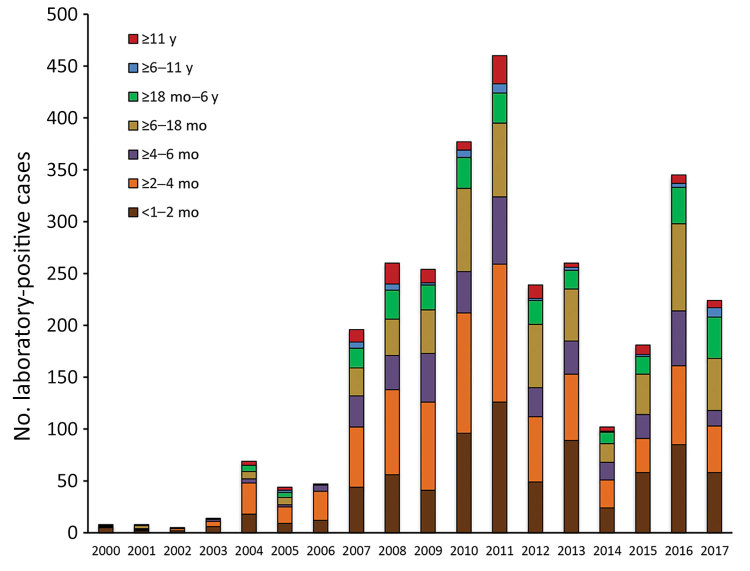
Number of laboratory-positive pertussis cases for 7 age cohorts, Buenos Aires, Argentina, 2000–2017.

The provincial cases per year distribution reflected the pattern of the entire country; 3 outbreaks were detected, in 2008, 2011, and 2016 (Figure 1). In each year of the period analyzed, most cases were detected in infants <1–2 months of age and those >2–4 months of age (Figure 2). The high proportion of cases recorded in patients <6 months of age was expected because pertussis is most severe in that age group.

We obtained the distribution of confirmed pertussis cases by patient age and vaccination status. Of confirmed cases, data were complete for 72.6% (2,338/3,220) of vaccinated persons and for 26.5% (619/2,338) of nonvaccinated persons <2 months of age. For infants <6 months of age, 45.3% had complete age-specific vaccination schedules. The percentage of patients with complete schedules was 53.7% for children >6 months of age and 6.4% for adolescents >11 years of age. Although this percentage for adolescents was low, this age group contained considerably fewer persons than those <6 months of age (44 infants vs. 1,590 children >6 months of age).

### *B. pertussis* Genotyping

**Figure 3:**
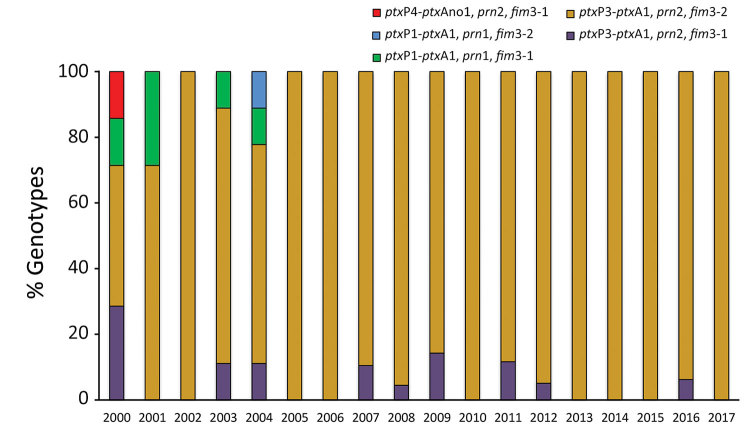
Percentage of multilocus sequence typing genotypes of *Bordetella pertussis* among isolates collected in Buenos Aires, Argentina, 2000–2017. *fim*, fimbriae; *prn*, pertactin; *ptx*A, pertussis toxin subunit A, *ptx*P, pertussis toxin promoter.

Almost all (99.7%) *B. pertussis* isolates analyzed contained the *ptx*A1 and *prn*2 alleles. Clinical isolates obtained during 2000–2004 had up to 4 different multilocus sequence typing genotypes (Figure 3). The index of discrimination calculated by year for this period ranged from 0.25 to 0.80. The highest value (higher diversity) was detected in 2000. The *ptx*P1 or *ptx*P4 variants were detected before 2004; thereafter, the *ptx*P3 locus prevailed. Most (291/350, 83.1%) isolates obtained after 2004 had the *ptx*P3-*ptx*A1-*prn*2-*fim*3–2 genotype. For 2004–2017, the index of discrimination ranged from 0 to 0.24, indicating the lowest diversity detected.

### Fimbriae Serotyping

Of 350 isolates tested, only 1 obtained during 2016 was classified as Fim2. The remaining isolates were classified as Fim3.

### PRN Immunoblots

Only 2 of the *B. pertussis* isolates included in this study were Prn deficient. Both strains were obtained from patients <1 year of age who had typical pertussis signs/symptoms. These 2 case-patients were linked in time (2016) but not geographically. One of these patients was born to a mother vaccinated with a PRN-containing aP vaccine and the other to a nonvaccinated mother. For these 2 strains, we detected insertion sequence 481 sequence (forward sense) at position 1613–1614 of *prn*, which disrupted the gene.

## Discussion

We conducted molecular genetic characterization of 350 *B. pertussis* isolates obtained during 2000–2017 from hospitalized patients in Buenos Aires, Argentina. Buenos Aires, similar to the entire country of Argentina, uses only wP vaccine for primary series of pertussis vaccinations. Most *B. pertussi*s isolates were obtained during the outbreaks detected during 2007–2008 (83), 2011–2012 (145), and 2016–2017 (45). A total of 78% of isolates were obtained from patients <6 months of age, 13.7% from patients 6–12 months of age, and 8.3% from patients >12 months of age. As expected, most *B. pertussis* isolates were from unvaccinated persons. As detected in other countries, we found that almost all isolates characterized had the Fim3 serotype (*35*).

Of 350 isolates, variants *ptx*P1 and *ptx*P4 and the allele *prn*1 were detected before 2004. Starting in 2004, a total of 313 isolates obtained had the *ptx*P3*-ptx*A1*-prn*2 alleles with either *fim*3–1 or *fim*–3-2. These genotypes differed from those of the vaccine-production strains (*36*) and were the most common in other countries that had vaccinated populations (*35*).

The polymorphism in PRN and subsequent spread of PRN-deficient isolates have elicited a deep concern in the healthcare system because these changes hypothetically might represent a selective avoidance by the bacteria of the immunity induced by the vaccines. The predominance of *prn*2 detected in more recent isolates from Buenos Aires is consistent with the hypothesis that strains in the vaccinated population with that allele are fitter than those harboring other *prn* alleles (*37*).

Regarding a deficiency in PRN expression, we detected only 2 isolates containing insertion sequence 481 in the coding region of *prn*. These isolates were obtained from infants <1 year of age that were linked in time (2016) but not location. One of these patients was born to a mother vaccinated with a PRN-containing aP vaccine and the other patient was born to a nonvaccinated mother.

There are no reports of pertactin deficiency in a country such as Argentina that includes wP vaccine as the only vaccine for primary pertussis vaccination. Recently, Poland, the only country in Europe that still uses the wP vaccine but also the aP vaccine for primary series, has reported detection of PRN-deficient clinical isolates (*38*). The percentage of those isolates was lower (15%) than that detected in the United States, Canada, or Australia (>65%), which only use aP vaccines (*10*,*39*,*40*). Detection of these isolates might be a consequence of the increase in the use of aP vaccines in Poland. Within this context, our study is apparently unique because Argentina uses only wP vaccine for the primary series of pertussis vaccinations.

This low frequency of PRN-deficient strains in regions where wP vaccine is still used supports the hypothesis that PRN-deficient clinical isolates have an advantage in aP vaccine–primed immunity (*41*). Accordingly, PRN-deficient clinical isolates were able to overcome an anti-PRN–mediated inhibition of macrophage cytotoxicity in vitro (*42*). Moreover, this study also showed that recently collected PRN-deficient *B. pertussis* clinical isolates harboring a *ptx*P3 variant and the *prn*2 allele had higher CFUs per lung and were capable of sustaining infection longer in aP vaccine–immunized mice than isolates still producing the protein. The investigators of that study speculated that these particular isolates might be capable of infecting immunized persons at an earlier stage of waning immunity after aP vaccine immunization or postinfection, thus having an advantage over isolates producing PRN. The findings of Hegerle et al. (*42*) are consistent with those recently reported by Safarchi et al. (*17*), which indicated a higher fitness of Prn-negative strains in aP vaccine–immunized mice. These investigators demonstrated in a mixed-infection model that PRN-negative *B. pertussis* colonized the respiratory tract of aP-immunized mice more effectively than the PRN-positive strain, thus outcompeting that strain (*17*).

Regarding a possible association between clinical findings and the PRN expression of the bacterial isolates that caused the human infections, recent studies suggest that symptoms (with the exception of apneas, which were less likely in PRN-deficient infections) and clinical course were similar regardless of PRN expression (*14*,*41*). Clarke et al. provide new data on this subject, which suggest that rapid emergence of PRN-deficient *B. pertussis* variants is unlikely to contribute to any greater risk for death or severe outcomes from infections in young, vulnerable infants (*43*).

Our study supports the hypothesis regarding pathogen adaptation of *B. pertussis* to the type of vaccine used. A key finding in this study was that use of the wP vaccine in the primary series of vaccinations correlated with a near complete absence of PRN-deficient strains, although the aP vaccine was used in subsequent vaccine regimens. Continued surveillance for PRN production in circulating *B. pertussis* is needed, as well as monitoring of other possible genotypic changes in the *B. pertussis* population, including a lack of expression of other immunogens contained in acellular vaccines.
